# Potential of prevention strategies for the modifiable risk factor type 2 diabetes with relation to the future number of dementia patients in Germany– a multi-state projection through 2040

**DOI:** 10.1186/s12883-022-02682-6

**Published:** 2022-04-26

**Authors:** Anne Fink, Achim Doerre, Ilja Demuth, Gabriele Doblhammer

**Affiliations:** 1 German Center for Neurodegenerative Diseases (DZNE), Bonn, Germany; 2 Robert Koch Institute, Department of Infectious Disease Epidemiology, Berlin, Germany; 3 Charité – Universitätsmedizin Berlin, corporate member of Freie Universität Berlin and Humboldt-Universität zu Berlin, Department of Endocrinology and Metabolic Diseases (including Division of Lipid Metabolism), Biology of Aging working group, Berlin, Germany; 4 Berlin Institute of Health at Charité – Universitätsmedizin Berlin, BCRT - Berlin Institute of Health Center for Regenerative Therapies, Berlin, Germany; 5 University of Rostock, Institute for Sociology and Demography, Rostock, Germany

**Keywords:** Diabetes, Dementia, Prevention strategies, Projection

## Abstract

**Background:**

We assess the impact of prevention strategies regarding type 2 diabetes as a modifiable risk factor for dementia and its consequences for the future number of dementia patients in Germany.

**Methods:**

We used a random sample of health claims data (*N* = 250,000) of insured persons aged 50+ drawn in 2014, and data on population size and death rates in 2015 from the Human Mortality Database. Using exponential hazard models, we calculated age- and sex-specific transition probabilities and death rates between the states (no diabetes/no dementia, diabetes/no dementia, no diabetes/dementia, diabetes/dementia). In multi-state projections, we estimated the future number of dementia cases aged 75+ through 2040 depending on the development of the incidence of diabetes among persons without diabetes and without dementia, and the dementia incidence among persons with and without diabetes.

**Results:**

In 2015 there were 1.53 million people with dementia aged 75+ in Germany. A relative annual reduction in death rates of 2.5% and in dementia incidence in persons without diabetes of 1% will increase this number to 3.38 million by 2040. A relative reduction of diabetes incidence by 1% annually would decrease dementia cases by around 30,000, while a reduction of dementia incidence among people with diabetes by 1% would result in 220,000 fewer dementia cases. Both prevention strategies combined would prevent 240,000 dementia cases in 2040.

**Conclusions:**

The increase in life expectancy is decisive for the future number of people with dementia. Strategies of better diabetes treatment have the potential to lower the increase in the number of dementia patients in the coming decades.

**Supplementary Information:**

The online version contains supplementary material available at 10.1186/s12883-022-02682-6.

## Background

Dementia is one of the most important diseases of old age in terms of number of patients, resulting care need, and costs [1, 2]. Despite the recent approval of aducanumab in the U.S., the focus remains on the prevention of dementia [3]. A large body of literature has shown the impact of modifiable risk factors on the incidence of dementia and their potential to prevent or at least to delay the onset of dementia and to postpone cognitive decline [4, 5]. The management or avoidance of these risk factors can be summarized as primary and secondary prevention strategies [6].

Diabetes mellitus is one major modifiable risk factor of dementia. Numerous studies have linked type 2 diabetes with an increased risk of cognitive impairment and dementia [7–9]. About 3 to 4% of all Alzheimer’s disease (AD) cases are attributable to diabetes [5], and diabetes patients have a 73% increased risk of any dementia, a 56% increase of AD, and a 127% increase of vascular dementia (VaD) [8]. The mechanisms of how diabetes is associated with impaired cognitive function are not yet fully clarified. In addition to hyper- and hypoglycemic conditions, the main contributors are decreased insulin secretion, obesity, increased oxidative stress, and inflammation [10]. Diabetes induces vascular pathologies such as stroke, which in turn are risk factors of dementia, especially VaD [7]. In addition to its contribution to vascular diseases, there is some evidence that diabetes may directly cause AD [10]. Because dementia starts developing 15 to 20 years prior to clinical symptoms, midlife diabetes is of particular importance. Longitudinal studies found a significantly increased risk of dementia for persons with midlife diabetes [11, 12].

In 2021, the global diabetes prevalence in adults aged 20 to 79 years was estimated to be 10.5%, in Europe diabetes prevalence was 9.5% [13], and a substantial proportion lives with undiagnosed diabetes [14]. Studies estimated that diabetes prevalence increased in the past decades [15–17], and projections for Germany indicate an additional increase [18].

A recent Cochrane review could not find definite proof for a special pharmacological therapy on the risk of cognitive decline and dementia [19]. However, some studies have indicated there is a protective effect of effective glycemic control via oral anti-diabetic medications on cognitive functioning [20, 21]. Furthermore, a review of diabetes-related dementia found promising evidence that regular physical activity and exercise might be beneficial for brain health in diabetes patients [22], and this is indicated by a number of studies (e.g. [23–25]). Espeland and colleagues found that a 24 months physical activity intervention was associated with improved performance of participants with diabetes in cognitive tests [26]. Additional evidence comes from animal experiments showing that exercise has positively impacted cognitive performance in a diabetes rat model [27]. However, additional research and especially randomized clinical trials in this field are undoubtedly warranted.

This study poses the question of whether preventing diabetes, or improving its treatment, has the potential to reduce the increasing number of dementia patients considerably through 2040. Preventing diabetes in mid-life may reduce the number of dementia patients in the long-term; better treatment of elderly diabetes patients may already have short to medium term effects by slowing down the progression rates to dementia. In order to answer this question, we try to quantify the effects of changes in the population structure (ageing of the baby boomers), increasing life expectancy, changes in diabetes incidence, and changes in the progression rates from diabetes to dementia, as well as from non-diabetes to dementia on the number of future dementia patients. We developed multi-state projections of the German population aged 75 and above through the year 2040, combining different assumptions about death rates and transition probabilities to depict possible effects of diabetes-prevention strategies, as well as improved diabetes-treatment options.

## Methods

### Human mortality database (HMD)

We used the Human mortality database (HMD, www.mortality.org), which provided information on the population size and death rates (50 to 110+ years) in Germany in the year 2015. In contrast to data from the Federal Statistical Office, the HMD provides information up to the highest ages.

### Health claims data

We used health claims data of the Allgemeine Ortskrankenkassen (AOK), the largest public health insurance company in Germany, to derive age- and sex-specific transition probabilities and mortality rate ratios. We used an age-stratified random sample of all AOK members in 2014 who had been born before 1965 (ages 50 years and above), which resulted in 250,000 insured persons; the sample was drawn by the data holder. Data were available until the end of 2017. The data consisted of demographic information on sex, date of birth, and date of death if applicable, as well as other information, and of medical information on all documented diagnoses and treatments of the inpatient and outpatient sectors. Diagnoses were coded by the German version of the 10th revision of the International Statistical Classification of Diseases and Related Health Problems (ICD-10-GM). All information was available on a quarterly basis. After data cleaning, we thus had information on 249,966 insured persons including 111,624 men and 138,342 women. Comparison against the total German population regarding age and sex show a good representativeness of the study population. Only the proportions in the highest age groups are larger than in the overall German population (see supplementary Fig. S1, Additional file 1). It is also known that AOK-insured persons have on average a lower socioeconomic status and higher morbidity rates than the general population [28]. This is also reflected in higher mortality rates.

### Definition of dementia

The following ICD-10 codes were used to define dementia: G30, G31.0, G31.82, G23.1, F00, F01, F02, F03, and F05.1. Because most dementia diagnoses are coded as “unspecified dementia” (F03), we do not differentiate by subtypes of dementia. Diagnoses in health claims data may be subject to legal changes or financial incentives. To overcome at least the problem of false-positive diagnoses [29], we applied a two-step validation strategy (see supplementary section “Validation of diagnoses”, Additional file 1). All subjects without a dementia diagnosis in 2014 and 2015 and a new diagnosis of dementia in 2016 or 2017 were assumed to be new cases.

### Definition of type 2 diabetes

In the data set type 2 diabetes was defined as having one of the ICD-10 codes E11-E14 assigned. All subjects without a valid diabetes diagnosis in 2014 and 2015 and a new diagnosis of diabetes in 2016 or 2017 were assumed to be new cases.

### Multi-state projection

**Fig. 1:**
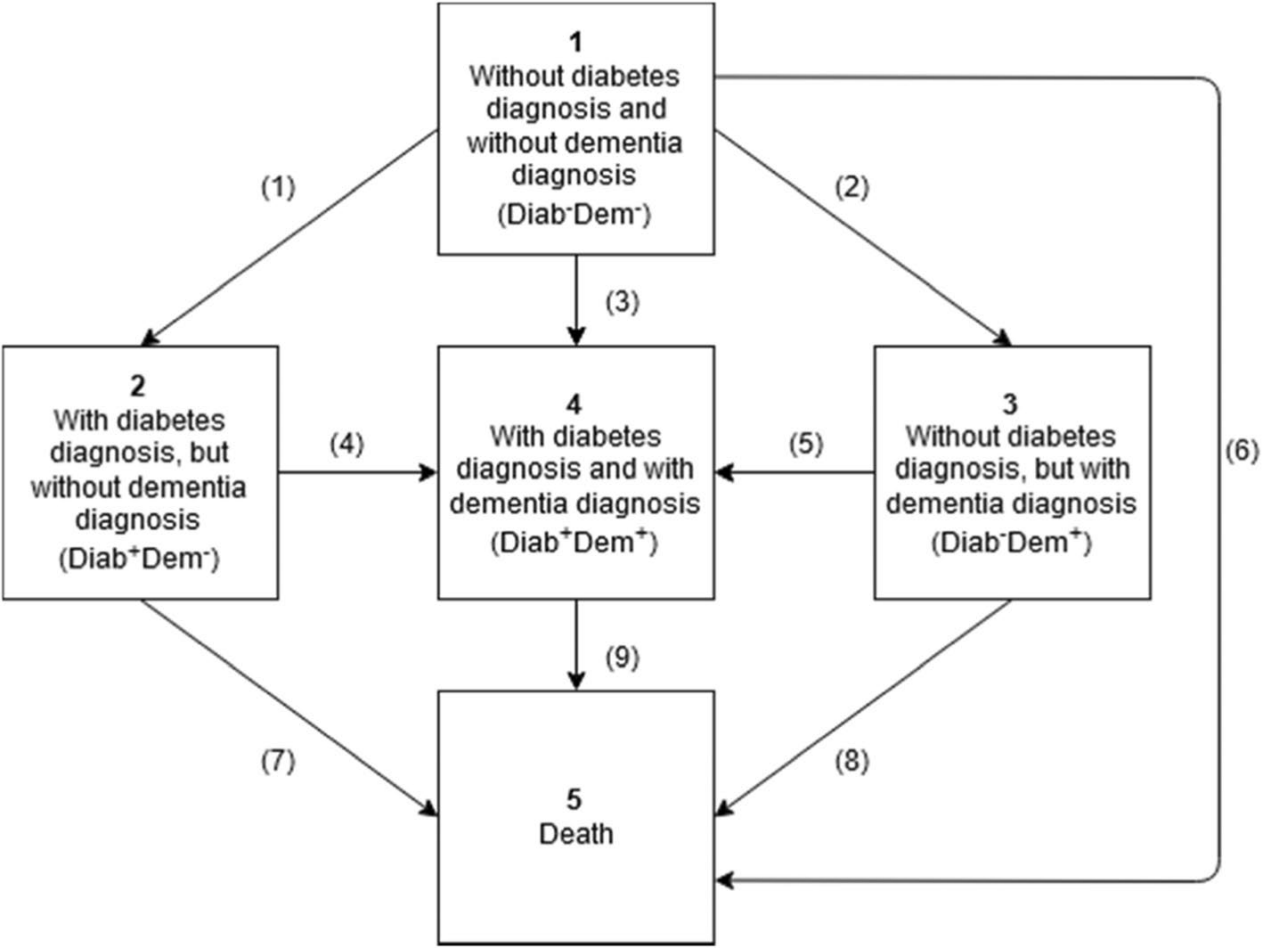
States and transitions for multi-state projection

To project the number of dementia patients aged 75 and above through the year 2040, we performed multi-state projections. We defined four transient states: 1. No diabetes / no dementia (Diab^−^Dem^−^), 2. Diabetes / no dementia (Diab^+^Dem^−^), 3. No diabetes / dementia (Diab^−^Dem^+^), 4. Diabetes / dementia (Diab^+^Dem^+^), and 5. Death as an absorbing state (Fig. 1). With transient states, there is the possibility of leaving the state but not returning, while absorbent states, once entered, cannot be left. From the AOK data, we calculated transition probabilities by age group and sex between the states. Because both dementia and diabetes are chronic diseases, transitions from diabetes to no diabetes and from dementia to no dementia rarely existed in the data and hence were not implemented in the multi-state model.

We explored dementia cases from age 75 onwards, so we did not consider fertility rates and assumed a closed population without migration from 2015 onwards. Over the projection horizon of 25 years, the starting population (aged 50 years and above in 2015) was exposed to the sex- and age-specific transition probabilities and death rates resulting in a population aged 75 and above by 2040. In order to assess the uncertainty of the multi-state projections for each scenario, we performed Monte Carlo simulation. In particular, we considered two main sources of uncertainty: First, the estimation of transition hazard rates which is subject to sampling error and second, the randomness of the actual transitions experienced by individuals during the projection horizon (see supplementary section “Multi-state projection” for a detailed description of the used methods, Additional file 1) .

Stata 16.0 was used for data management and regression analysis. The algorithm for performing the simulations has been implemented in R version 4.0.2 and RStudio version 1.3.1073.

### Calculation of death rates *m*_*x,t*_

To calculate the death rates *m*_*x,t*_, we estimated age- and sex-specific mortality rate ratios of the four states based on AOK data. Therefore, we performed exponential hazard models for the risk of death separately by sex for the whole study population and for the four states separately with age and the quadratic term of age as explaining variables.

Age- and sex-specific mortality rate ratios were calculated by dividing predicted age- and- sex-specific mortality rates of the four states by predicted age- and- sex-specific mortality rates of the total study population. The resulting age- and- sex-specific mortality rate ratios were multiplied with HMD death rates to obtain state-specific death rates ${m}_{x,t}^s$ calibrated to the level of the total German population.

### Calculation of the transition probabilities 𝑡𝑟

First, age-specific hazard rates for the transitions (1) to (5) were derived separately by sex from exponential hazard models with age as well as the quadratic term of age as explaining variables. We assumed an exponential baseline hazard because the time trend in dementia risk over a three-year period is negligible. We used the predicted age-specific hazard rates ${h}_{x,t}^s$ and age-specific death rates ${m}_{x,t}^s$ to calculate separate age-specific transition probabilities ${tr}_{x,t}^{s,s}$ for men and women (see supplementary section “Calculation of transition probabilities”, Additional file 1).

### Variants of assumptions

We modeled different scenarios with assumptions about the development of the transition probabilities and death rates. To explore the effect of changes in the age structure, we developed Scenario 1 (Sc1) with constant death rates and transition probabilities. We compared Sc1 with Scenario 2 (Sc2) to demonstrate the effect of increasing life expectancy. Here, we assumed a relative reduction of annual death rates in all states by 2.5%. This assumption is comparable with modeled mortality reductions in the 14th coordinated population projection of the German Federal Statistical Office [30]. Scenario 3.0 (Sc3.0) additionally modeled general unspecific dementia prevention strategies regarding the population without diabetes and dementia. Here, we assumed a relative reduction of age-specific dementia incidence by 1% annually. The Alzheimer Cohorts Consortium recently reported a 13% decline per decade in dementia incidence [31], which corresponds to a 1% reduction per year.

Under the assumption of an increasing life expectancy and decreasing dementia incidence as modeled in Sc3.0, we approximated strategies of primary and secondary prevention regarding diabetes. Firstly, we incorporated changes only in one transition to explore the single effect of a prevention strategy on the number of future dementia cases (Scenarios 3.1 and 3.2). Subsequently, we combined prevention strategies (Scenarios 3.3).

Scenario 3.1 (Sc3.1) 1 Diab^−^Dem^−^ ➔ 2 Diab^+^Dem^−^: To model the impact of a decreasing incidence of diabetes on the future number of dementia cases, we assumed a relative reduction of annual diabetes incidence of persons without dementia by 1%. All other transitions did not change. In fact, data on temporal trends of diabetes incidence in Germany are limited [32]. However, since we would like to model prevention strategies a hypothetical annual reduction of 1% was assumed.

Scenario 3.2 (Sc3.2) 2 Diab^+^Dem^−^ ➔ 4 Diab^+^Dem^+^: Improvements in the treatment of diabetes regarding cognitive outcomes were accounted for by a relative reduction of annual age-specific dementia incidence of diabetes patients by 1% as well. Since the magnitude effect of a successful diabetes treatment in relation to dementia is not yet fully understood, we again assumed a hypothetical annual decrease in dementia incidence of 1% for persons with diabetes.

Scenario 3.3 (Sc3.3) 1 Diab^−^Dem^−^ ➔ 2 Diab^+^Dem^−^ and 2 Diab^+^Dem^−^ ➔ 4 Diab^+^Dem^+^: We combined both prevention strategies regarding diabetes and simultaneously assumed an annual relative reduction in diabetes incidence of 1% in persons without diabetes and dementia and a relative reduction of dementia incidence of 1% per year in persons with diabetes.

## Results

In the base year 2015, there were 9 million people aged 75 years and above in Germany. At age 75, 7.4% of men and 7.7% of women were affected by dementia. This share increased steeply with age, and in the highest age group aged 89 years and above 30.8% of men and 40.0% of women had a dementia diagnosis.

According to the prevalences from the AOK data (Table 1), 4.99 million people of this age group had neither a diagnosis of diabetes nor a diagnosis of dementia (Diab^−^Dem^−^); 2.47 million people had been diagnosed with diabetes, but without dementia (Diab^+^Dem^−^); 0.94 million people had been diagnosed as dementia patients without diabetes (Diab^−^Dem^+^); and 0.59 million people had a diagnosis of diabetes and dementia (Diab^+^Dem^+^). In total, 1.53 million people had received a dementia diagnosis (with and without diabetes diagnosis) in 2015.

**Table 1:** Results of multi-state projection, estimated number of persons by state in Germany aged 75+ in 2040, 95% uncertainty intervals in brackets

			Projected number by state in million									
**Year**	**Scenario**	**Assumptions**	Total	Diab^−^Dem^−^	Diab^+^Dem^−^	Diab^−^Dem^+^	Diab^+^Dem^+^	**Total dementia cases**	Total diabetes cases	Change in Diab^−^Dem^+^ cases compared to Scenario 3.0	Change in Diab^+^Dem^+^ cases compared to Scenario 3.0	Change in total dementia cases compared to Scenario 3.0
**2015**	**Base year**		9.00	4.99	2.47	0.94	0.59	**1.53**	3.07			
**2040**	**Scenario 1**	**Status quo**	11.55 (11.22–11.88)	5.97 (5.70–6.24)	3.36 (3.30–3.41)	1.03 (1.00–1.06)	1.20 (1.17–1.22)	**2.23 (2.20–2.25)**	4.55 (4.49–4-61)	–	–	–
	**Scenario 2**	**Death rates by − 2.5%**	14.58 (14.24–14.90)	6.85 (6.57–7.15)	4.09 (4.03–4.16)	1.61 (1.57–1.64)	2.03 (1.97–2.09)	**3.63 (3.59–3.69)**	6.12 (6.04–6.22)	–	–	–
	**Scenario 3.0**	**Death rates by − 2.5%, dementia incidence of Diab**^**−**^**Dem**^**−**^ **by − 1%**	14.70 (14.36–15.01)	7.20 (6.91–7.50)	4.12 (4.06–4.19)	1.38 (1.35–1.41)	2.00 (1.94–2.05)	**3.38 (3.33–3.43)**	6.12 (6.04–6.21)	–	–	–
	**Scenario 3.1**	**Death rates by − 2.5%, dementia incidence of Diab**^**−**^**Dem**^**−**^ **by − 1%, diabetes incidence of Diab**^**−**^**Dem**^**−**^ **by − 1%**	14.73 (14.40–15.05)	7.59 (7.30–7.89)	3.80 (3.74–3.86)	1.43 (1.40–1.46)	1.92 (1.87–1.97)	**3.35 (3.30–3.40)**	5.72 (5.64–5.81)	+ 3.5% (+ 3.3% − + 3.8%)	−3.9% (− 4.1%--3.8%)	−0.9% (− 1.0%--0.8%)
	**Scenario 3.2**	**Death rates by − 2.5%, dementia incidence of Diab**^**−**^**Dem**^**−**^ **by − 1%, dementia incidence of Diab**^**+**^**Dem**^**−**^ **by − 1%**	14.80 (14.46–15.11)	7.20 (6.91–7.50)	4.44 (4.37–4.51)	1.38 (1.35–4.41)	1.78 (1.73–1.83)	**3.16 (3.12–3.21)**	6.22 (6.13–6.32)	0.0% (− 0.1% − + 0.1%)	−10.9% (− 11.1%--10.6%)	−6.4% (− 6.5%--6.3%)
	**Scenario 3.3**	**Death rates by − 2.5%, dementia incidence of Diab**^**−**^**Dem**^**−**^ **by − 1%, diabetes incidence of Diab**^**−**^**Dem**^**−**^ **by − 1%, dementia incidence of Diab**^**+**^**Dem**^**−**^ **by − 1%**	14.83 (14.50–15.14)	7.59 (7.30–7.89)	4.10 (4.03–4.17)	1.43 (1.40–1.46)	1.71 (1.67–1.77)	**3.14 (3.10–3.19)**	5.81 (5.73–5.91)	+ 3.5% (+ 3.3% − + 3.8%)	− 14.1% (− 14.3%--13.8%)	−6.9% (− 7.0%--6.8%)

Note: Source: AOK 2014–2017 and HMD

### Estimated transition probabilities

**Fig. 2:**
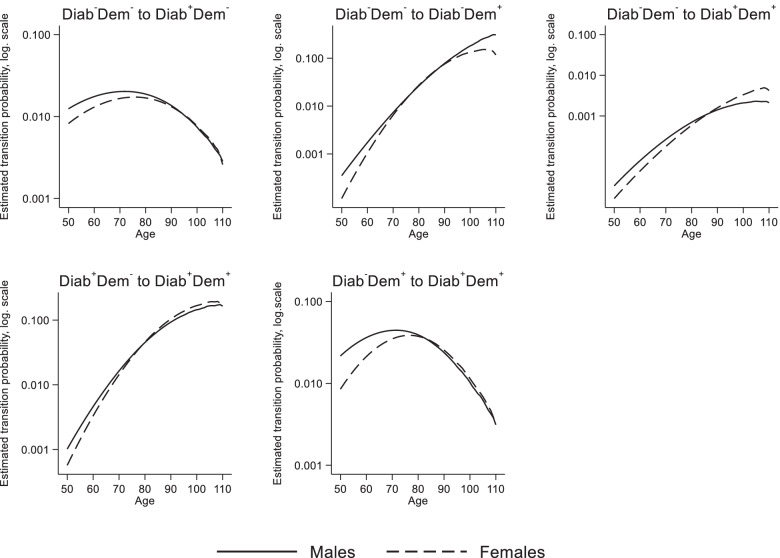
Estimated transition probabilities (logarithmic scale) by sex and age. Note: Diab^−^Dem^−^: persons without a valid diabetes diagnosis and without a valid dementia diagnosis, Diab^+^Dem^−^: persons with a valid diabetes diagnosis, but without a dementia diagnosis, Diab^−^Dem^+^: persons with a valid dementia diagnosis, but without a diabetes diagnosis, Diab^+^Dem^+^: persons with a valid diabetes diagnosis and a valid dementia diagnosis. Source: AOK 2014–2017

Figure 2 shows the estimated age- and sex-specific transition probabilities for the first year 2015. At age 50, about 1.2% of men and 0.8% of women of state 1 (Diab^−^Dem^−^) received a diabetes diagnosis within one year. Probabilities increase with age up to age 72 for men (2.0%) and 76 for women (1.7%); thereafter the incidence of diabetes decreases for both sexes. For the transition from state 1 (Diab^−^Dem^−^) to state 3 (Diab^−^Dem^+^) we observed an exponential increase for men and women, with slightly higher rates for men than for women. At age 50, the probability to transit was 0.04% for men and 0.01% for women, at age 90 it was 7.7% for men and 7.5% for women. The probability to experience the transition to diabetes and dementia at the same time was very low (state 1 to state 4). From age 80 onwards this probability was between 0.1 0.2% for men and women.

Regarding the transition from state 2 (Diab^+^Dem^−^) to state 4 (Diab^+^Dem^+^), we observed an exponential increase for men and women, with slightly higher rates for men than for women until the age of 78. At age 50, the probability was 0.1%, and at age 90, probabilities were 9.3% for men and 10.4% for women. Up to age 94 for men and at all ages for women, the dementia probability of persons with diabetes was higher than for persons without diabetes. For the transition from state 3 (Diab^−^Dem^+^) to state 4 (Diab^+^Dem^+^), we observed again a reverse U-shaped pattern with a probability of 4.4% for men at age 72, and 3.9% for women at age 77.

### Estimated death rates

**Fig. 3:**
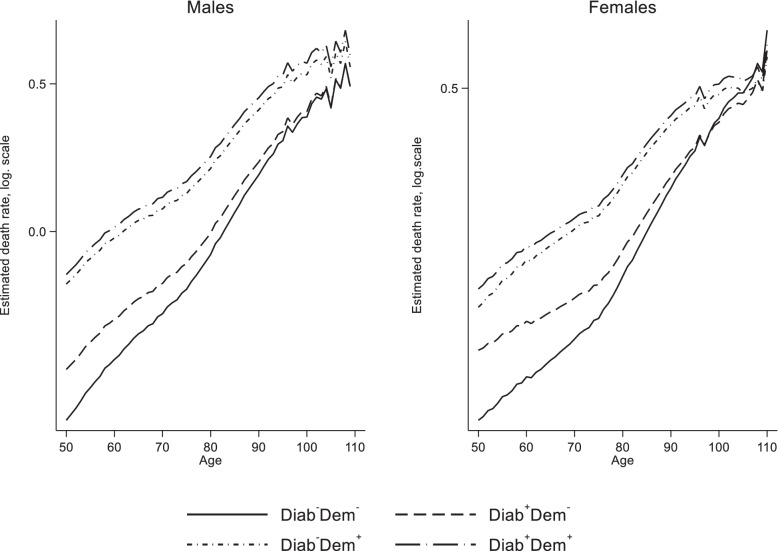
Estimated death rates (logarithmic scale) from exponential hazard model by sex, age and state. Note: Diab^−^Dem^−^: persons without a valid diabetes diagnosis and without a valid dementia diagnosis, Diab^+^Dem^−^: persons with a valid diabetes diagnosis, but without a dementia diagnosis, Diab^−^Dem^+^: persons with a valid dementia diagnosis, but without a diabetes diagnosis, Diab^+^Dem^+^: persons with a valid diabetes diagnosis and a valid dementia diagnosis. Source: AOK 2014–2017 and HMD

Death rates (Fig. 3) rise exponentially with age, and individuals without diabetes and dementia (Diab^−^Dem^−^) had the lowest death rates at all ages, followed by those with diabetes only (Diab^+^Dem^−^) and dementia only (Diab^−^Dem^+^). Patients with diabetes and dementia had the highest death rates. For example, at age 80, the death rate of men of state 1 (Diab^−^Dem^−^) was 0.04, men of state 2 (Diab^+^Dem^−^) had 0.05, men of state 3 (Diab^−^Dem^+^) had 0.14 and the death rate of men with diabetes and dementia (Diab^+^Dem^+^) was 0.17. For women, corresponding values were 0.02, 0.04, 0.10 and 0.12 (Fig. 3).

### Results of multi-state projections

Table 1 presents the results of the multi-state projections with means and 95% uncertainty interval in brackets. Modeling the demographic components of the future number of dementia patients reveals the overwhelming effect of future increases in life expectancy. Starting with 9 million individuals at ages 75 and above in 2015, the ageing of the baby boomer generation will lead to an increase of up to 11.55 million in 2040, even under the assumption of constant transition probabilities and death rates (Sc1, Table 1). Rising life expectancy, modeled by a relative annual reduction of death rates in all states by 2.5%, results in 14.58 million people (Sc2), which is comparable to the results of the 14th coordinated population projection of the Federal Statistical Office in Germany [30].

**Fig. 4:**
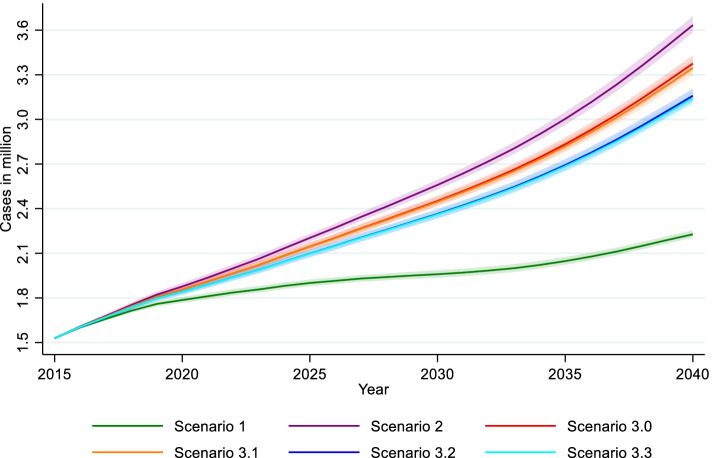
Results of multi-state projection by scenarios, estimated number with 95% uncertainty interval of total dementia cases aged 75+ through 2040. Note: Scenario 1: Status quo, scenario 2: Death rates by − 2.5%, scenario 3.0: Death rates by − 2.5%, dementia incidence of Diab^−^Dem^−^ by − 1%, scenario 3.1: Death rates by − 2.5%, dementia incidence of Diab^−^Dem^−^ by − 1%, diabetes incidence of Diab^−^Dem^−^ by − 1%, scenario 3.2: Death rates by − 2.5%, dementia incidence of Diab^−^Dem^−^ by − 1%, dementia incidence of Diab^+^Dem^−^ by − 1%, scenario 3.3: Death rates by − 2.5%, dementia incidence of Diab^−^Dem^−^ by − 1%, diabetes incidence of Diab^−^Dem^−^ by − 1%, dementia incidence of Diab^+^Dem^−^ by − 1%. Source: AOK 2014–2017 and HMD

Under the assumption of constant transition probabilities and death rates (Sc1) there will be a growth in the absolute number of persons with dementia, from 1.53 million in 2015 to 2.23 million in 2040, corresponding to an increase of 46% compared to 2015. Thus, the ageing of the baby boomers alone will add about 700,000 dementia patients. Relative annual reductions of death rates by 2.5% will additionally add 1.4 million dementia patients, totaling up to 3.63 million affected persons in 2040 (Sc2, Table 1 and Fig. 4). In Sc3.0, a relative reduction of 1% per year in dementia incidence in persons without diabetes and dementia yields 3.38 million total dementia cases in 2040 (3.38 million in Sc3.0–3.63 million in Sc2 = − 250,000, − 6.9%).

In the following, we use the projections for 2040 from Sc3.0 as reference values.

In Sc3.1 (Table 1, Fig. 4), the 1% relative annual reduction in diabetes incidence results in a reduced number of dementia cases with diabetes (Diab^+^Dem^+^: 1.92 million in Sc3.1–2.00 million in Sc3.0 = − 80,000, − 3.9%), while dementia cases without diabetes (Diab^−^Dem^+^) increase by + 3.5% (1.43 million in Sc3.1–1.28 million in Sc3.0 = + 50,000). This is because more people remain in state 1 under this assumption and thus are at risk of dementia. In sum, this strategy only slightly affects the future total number of dementia cases with 30,000 fewer cases (3.35 million in Sc3.1–3.38 million in Sc3.0 = − 30,000, − 0.9%).

In Sc3.2, a relative reduction of 1% per year in the incidence of dementia in diabetes patients was assumed (Table 1, Fig. 4). This results in 1.78 million dementia cases with diabetes (Diab^+^Dem^+^: 1.78 million in Sc3.2–2.00 million in Sc3.0 = − 220,000, − 10.9%), while the trend in the number of dementia cases without diabetes (Diab^−^Dem^+^) remains unchanged. Overall, this strategy results in 220,000 fewer dementia cases (3.16 million in Sc3.2–3.38 million in Sc3.0 = − 220,000, − 6.4%).

Sc3.3 combines the two prevention strategies. Again, we observe a + 3.5% increase in dementia cases without diabetes (Diab^−^Dem^+^) (1.43 million in Sc3.3–1.38 million in Sc3.0 = + 50,000), but a 290,000 decrease in the number of individuals with both diagnoses to 1.71 million cases (Diab^+^Dem^+^: 1.71 million in Sc3.3–2.00 million in Sc3.0 = − 290,000, − 14.1%). Combining the two prevention strategies yields 3.14 million dementia cases (3.14 million in Sc3.3–3.38 million in Sc3.0 = − 240,000, − 6.9%).

## Discussion

By 2040, the future number of dementia patients aged 75 years and above will be mainly driven by increases in life expectancy and changes in the age structure as a result of past fertility patterns. One third of the increase in dementia cases is due to changes in the age structure (Sc1), while two thirds of the increase is due to increasing life expectancy (Sc2). This increase can be slowed with about 250,000 fewer dementia cases in 2040 if we assume a continuation of past time trends with a 1% annual reduction in dementia incidence (Sc3.0), as reported by Alzheimer Cohorts Consortium [31].

When modeling the putative effect of interventions in the context of diabetes with a focus on dementia incidence, two prevention strategies can be pursued. First, the incidence of diabetes can be reduced, second the incidence of dementia in diabetes patients can be reduced. The first strategy is depicted in Sc3.1. However, with 30,000 fewer dementia patients, the effect is marginal. This is the counterbalancing result of an increase in dementia patients without diabetes and a decrease in dementia patients with diabetes. Detrimental consequences of diabetes on cognition only accumulate over decades [11, 12], so that primary disease prevention measures only have their full protective effect in the long term and are not able to compensate for the baby boomer effect and the even much larger effect of increasing life expectancy in the medium term. Mukadam et al. [33] concluded that preventing diabetes would not be cost-effective because of its impact on dementia alone. Although the impact of a decrease in diabetes incidence on the number of dementia cases is quite small, a decrease in diabetes incidence would have many other benefits. For example, as shown in Fig. 3, mortality in the dementia population would be lower because there would be fewer people suffering from both dementia and diabetes.

The second strategy is to reduce the incidence of dementia (Sc3.2) in diabetes patients, which would have a sizeable and direct effect on the number of dementia patients. About 220,000 dementia patients could be prevented. Effective blood glucose control via diet, exercise and anti-diabetic medication may be key to these reductions [20–22, 34]. Combined implementation of both prevention strategies (Sc3.3) could prevent 240,000 dementia patients in 2040.

Here, we only present the possible impact of one modifiable risk factor for dementia. In fact, the presence of diabetes is not independent from other potentially modifiable risk factors such as hypertension, physical inactivity, obesity, smoking, depression, or low educational attainment which are in turn also associated with dementia. Norton et al. showed that in Europe about 3.1% of all AD cases were attributable to diabetes. For comparison, more than 20% of all AD cases were attributable to physical inactivity [5]. As we have no information on socio-economic and life style factors in our data, we cannot assess the impact of these factors on dementia.

Compared to other dementia projections for Germany, our results are at the upper boundaries. A recent study based on AOK data for Baden-Wurttemberg estimated there would be 1.8 to 2.9 million dementia cases for the total population in 2040 [35]. Baden-Wurttemberg is a federal state in Germany with one of the highest life expectancy rates and lowest morbidity. We used a random sample of all persons insured with the AOK in Germany, which may lead to higher dementia estimates. Another study based on meta-data of several population surveys projected there will be 2.4 to 2.6 million dementia cases aged 65+ in 2040 [36]. However, this study assumed time-constant prevalence rates.

### Strengths and limitations

The present study has several strengths and limitations. Our analyses rely on a high case number so that we can estimate transition probabilities and death rates up to the highest ages. Because of the routine documentation by physicians and hospitals, bias due to non-response, forgetfulness, interviewer bias, or self-selection can be ruled out. The institutionalized population is included regardless of their functional and cognitive status, which is particular important when analyzing dementia.

Health claims data are used primarily for billing purposes in health care and are not created for epidemiological analyzes. Therefore, documented diagnoses inevitably cannot reflect epidemiological disease development at the population level. First, persons who do not consult a doctor are not included in the diagnoses data. Second, coding errors and false-positive diagnoses are possible. To counteract this bias, we use an established two-stage validation procedure for dementia and diabetes.

While AOK claims data cover the total German population, the proportions of people with lower incomes and low education levels is higher than in other public health insurers in Germany, which leads to higher morbidity rates [28]. It is thus possible that these results may not be truly general for the total German population.

### Implication for public health

There will be a marked increase of persons with dementia within the next 20 years in Germany. We have shown the potential that the prevention and the better treatment of the modifiable risk factor type 2 diabetes might have to counterbalance some of this increase. There is a great deal of evidence that diabetes patients do not always adhere to treatment in terms of anti-diabetic medications or recommendations regarding diet and physical activity [37–40]. This holds true despite the fact that early detection of diabetes and subsequent treatment are essential, as the risk of dementia increases with the progression of diabetes [41]. Furthermore, only about half of the population without diabetes considers themselves to be well-informed about the causes and consequences of diabetes [42], which would be another important prerequisite for the primary prevention of diabetes with positive long-term consequences on the number of dementia patients.

From a societal point of view, reducing dementia is central because the illness is very care-intensive and therefore cost-intensive. There is a long period of care dependency over the course of the illness, as dementia patients spend 30% of their remaining life expectancy in the moderate stage of the illness, and 40% in the most severe stage of the illness [43]. Compared to previous cohorts, the baby boomer generation on average had fewer children who could provide care for parents. Thus, the future demographic development of the German population implies there will be an increasing imbalance between the number of potential caregivers and people with need for care [44].

## Conclusion

Our results emphasize the need for prevention strategies of modifiable risk factors in order to lower the future number of dementia patients in Germany. We have focused on type 2 diabetes, which is only one major modifiable risk factor of dementia. If we succeed in reducing the incidence of additional risk factors such as hypertension, smoking, low education, or physical inactivity, then the reduction of the increase of the future number of dementia patients might be even higher. As most modifiable risk factors also contribute to other major diseases of the elderly, not only would dementia be prevented, but also cardiovascular disease and subsequent need for care and medical services. More research is needed to evaluate the effectiveness of prevention strategies and tailor these to the needs of the different strata of the population.

## Supplementary Information

**Additional file 1**

## Data Availability

Human Mortality Database (HMD). All data are publicly available from the Human Mortality Database (www.mortality.org). Health claims data. The scientific research institute of the AOK (WIdO) has strict rules regarding data sharing because of the fact that health claims data are a sensitive data source and have ethical restrictions imposed due to concerns regarding privacy. Anonymized data are available to all interested researchers upon request. Interested individuals or an institution who wish to request access to the health claims data of the AOK may contact the WIdO (webpage: http://www.wido.de/, mail: wido@wido.bv.aok.de).

## Abbreviations

AD: Alzheimer’s disease
AOK: Allgemeine Ortskrankenkassen
Diab−Dem−: No diabetes / no dementia
Diab^+^Dem^−^: Diabetes / no dementia
Diab^−^Dem^+^: No diabetes / dementia
Diab^+^Dem^+^: Diabetes / dementia
HMD: Human Mortality Database
ICD: International Statistical Classification of Diseases and Related Health Problem
Sc: Scenario
VaD: Vascular dementia
