# Supplementary material for: Potential of prevention strategies for the modifiable risk factor type 2 diabetes with relation to the future number of dementia patients in Germany– a multi-state projection through 2040
Source: BMC Neurol. 2022 Apr 26;22:157. doi: 10.1186/s12883-022-02682-6 (PMC9040288; doi:10.1186/s12883-022-02682-6)
Supplement: Supplementary file 1 — Additional file 1 [file 12883_2022_2682_MOESM1_ESM.docx]

Potential of prevention strategies for the modifiable risk factor type 2 diabetes with relation to the future number of dementia patients in Germany– A multi-state projection through 2040

Authors: Anne Fink^1^, PhD; Achim Doerre^2^, PhD; Ilja Demuth^3,4^, Prof. & Gabriele Doblhammer^1,5^, Prof.

^1^ German Center for Neurodegenerative Diseases (DZNE), Bonn, Germany

^2^ Robert Koch Institute, Department of Infectious Disease Epidemiology, Berlin, Germany

^3^ Charité – Universitätsmedizin Berlin, corporate member of Freie Universität Berlin and Humboldt-Universität zu Berlin, Department of Endocrinology and Metabolic Diseases (including Division of Lipid Metabolism), Biology of Aging working group, Berlin, Germany

^4^ Berlin Institute of Health at Charité – Universitätsmedizin Berlin, BCRT - Berlin Institute of Health Center for Regenerative Therapies, Berlin, Germany

^5^ University of Rostock, Institute for Sociology and Demography, Rostock, Germany

**SUPPLEMENTAL MATERIAL**

Figure S1: Composition of study population (AOK) and total German population (HMD) by sex and age. Source: AOK 2014-2017 and HMD.

**Validation of diagnoses**

1. Validation strategy of dementia diagnoses

First, from the inpatient sector only discharge or secondary diagnoses were considered. Outpatient diagnoses had to be marked with the modifier “verified”. Second, diagnoses were defined as valid if they appeared simultaneously in both the inpatient and outpatient sectors, or if two different types of physicians each made the diagnosis in the same quarter. Furthermore, a diagnosis could be confirmed by a second occurrence over the course of the analysis period. If a person died in the same quarter the first diagnosis was made, that diagnosis was considered to be valid even if there was no confirmative diagnosis.

1. Validation strategy of diabetes diagnoses

We only considered inpatient discharge or secondary diagnoses and outpatient diagnoses which were marked as verified. A diabetes diagnosis was assumed to be valid if the diagnosis could be confirmed by a second one, or if the diagnosis occurred in the quarter of death.

**Multi-state projection**In order to assess the uncertainty of the projections for each scenario, we perform Monte Carlo simulation. In particular, we consider two main sources of uncertainty: First, the estimation of transition hazard rates which is subject to sampling error and second, the randomness of the actual transitions experienced by individuals during the projection horizon.

To incorporate the first, we perform random draws from the approximate multivariate normal distribution of regression coefficients obtained from the exponential hazards model for each transition. Each random draw represents one hypothetical true constellation of regression coefficients and hence the corresponding transition hazards. It is noted that the variance-covariance matrix of the multivariate normal distribution incorporates both the standard errors and pairwise correlations of the estimated regression coefficients, and therefore the hazard parameters of each transition are not treated as independent.

To incorporate the second source of uncertainty, transitions between states are modeled stochastically based on the respective transition hazards. In detail, we model the age- and sex-specific number of individuals in each state, and at each discrete time step covering one year, individuals may either stay in their current state or move to an adjacent one. Within every time step, at most one transition to another state is possible, and the probability of staying in a state is given by the survival distribution of moving to any other adjacent state. The transitions are modeled stochastically for each individual using a multinomial distribution with the transition probabilities derived via numerical integration from the transition hazards. In calculating the age-specific transition probabilities, the competing risk aspect in case of several adjacent states is incorporated.

For each scenario, we perform 1000 simulation runs each consisting of 25 time steps according to the described scheme. Based on the resulting hypothetical projections, we determine uncertainty intervals for all aspects of interest, including e.g. the overall number of people within the separate states or the overall number of people with dementia.

It is noted that the described simulation approach yields uncertainty intervals which reflect the uncertainty regarding the transition hazards estimates and randomness of realized transition paths. These intervals are not to be confused with conventional confidence intervals of the projected aspects of interest, as these would rather relate to actual forecasts and require a different set of information and assumptions. The main purpose of our investigation, however, is to assess the potential impact of changes in the overall mortality, diabetes and dementia rates, as represented by the scenarios.

The algorithm for performing the simulations has been implemented in R version 4.0.2 and RStudio version 1.3.1073. To evaluate the correctness of the algorithm, the results have been checked with a simpler deterministic projection method using the point estimates of the transition hazard regression models.

**Calculation of transition probabilities**

We calculated the probability to stay in state 1 (Diab^-^Dem^-^) ${tr}_{x,t}^{1,1}$ for sex *x* and age *t*, for example, by

${tr}_{x,t}^{1,1}=\exp({-M}_{x,t}^{1}-{(H}_{x,t}^{1,2}+H_{x,t}^{1,3}+H_{x,t}^{1,4}))$ ,

where

$$M_{x,t}^{j}=\int_{t}^{t+1} m_{x,t}^{j} dt{\mathrm{and} H}_{x,t}^{j,k}=\int_{t}^{t+1} h_{x,t}^{j,k} dt$$

are the cumulative hazard functions, defined for any states *j* and *k*. The probability to transit from state 1 (Diab^-^Dem^-^) to state 2 (Diab^+^Dem^-^), for example, was then calculated by

$${tr}_{x,t}^{1,2}=\left( 1-{tr}_{x,t}^{1,1} \right)\left( \frac{H_{x,t}^{1,2}}{{M_{x,t}^{1}+H}_{x,t}^{1,2}+H_{x,t}^{1,3}+H_{x,t}^{1,4}} \right),$$

which is the product of the probability of leaving state 1, i.e. $1-{tr}_{x,t}^{1,1}$, and the relative contribution of the cumulative hazard function $H_{x,t}^{1,2}$ to the overall hazard function of any transition from state 1. Transition probabilities for other states are determined with according changes depending on the respective adjacent states.
